# Functionalization of the NiTi Shape Memory Alloy Surface through Innovative Hydroxyapatite/Ag-TiO_2_ Hybrid Coatings

**DOI:** 10.3390/ma17030604

**Published:** 2024-01-26

**Authors:** Karolina Dudek, Mateusz Dulski, Jacek Podwórny, Magdalena Kujawa, Anna Gerle, Patrycja Rawicka

**Affiliations:** 1 Łukasiewicz Research Network–Institute of Ceramics and Building Materials, Cementowa 8, 31-983 Kraków, Poland; jacek.poworny@icimb.lukasiewicz.gov.pl (J.P.); magdalena.kujawa@icimb.lukasiewicz.gov.pl (M.K.); anna.gerle@icimb.lukasiewicz.gov.pl (A.G.); 2 Institute of Materials Engineering, University of Silesia, 75 Pułku Piechoty 1A, 41-500 Chorzów, Poland; 3 Institute of Physics, Faculty of Science and Technology, University of Silesia in Katowice, 75 Pułku Piechoty 1a, 41-500 Chorzów, Poland; patrycja.rawicka@us.edu.pl

**Keywords:** silver-rutile Ag-TiO_2_ nanocomposite, hydroxyapatite (HAp), surface modification, Raman spectroscopy

## Abstract

The objective of this research was to develop a surface modification for the NiTi shape memory alloy, thereby enabling its long-term application in implant medicine. This was achieved through the creation of innovative multifunctional hybrid layers comprising a nanometric molecular system of silver-rutile (Ag-TiO_2_), known for its antibacterial properties, in conjunction with bioactive submicro- and nanosized hydroxyapatite (HAp). The multifunctional, continuous, crack-free coatings were produced using the electrophoretic deposition method (EPD) at 20 V/1 min. Structural and morphological analyses through Raman spectrometry and scanning electron microscopy (SEM) provided comprehensive insights into the obtained coating. The silver within the layer existed in the form of nanometric silver carbonates (Ag_2_CO_3_) and metallic nanosilver. Based on DTA/TG results, dilatometric measurements, and high-temperature microscopy, the heat treatment temperature for the deposited layers was set at 800 °C for 2 h. The procedures applied resulted in the creation of a new generation of materials with a distinct structure compared with the initial nanopowders. The resulting composite layer, measuring 2 μm in thickness, comprised hydroxyapatite (HAp), apatite carbonate (CHAp), metallic silver, silver oxides, Ag@C, and rutile exhibiting a defective structure. This structural characteristic contributes significantly to its heightened activity, influencing both bioactivity and biocompatibility properties.

## 1. Introduction

Within the spectrum of calcium phosphate ceramics (CaPs) widely applied in regenerative medicine, particularly in orthopedics, significant attention is directed towards hydroxyapatite (HAp). HAp belongs to the group of biomaterials characterized by high biocompatibility with bone tissue, high compressive strength, hardness (similar to tissue), biotolerance, and corrosion resistance, thus ensuring cell proliferation, bioactivity, etc. Consequently, calcium phosphate-based ceramics are often applied to create coatings on metallic implants through diverse surface modification methods. Yet, despite its numerous advantages, pure apatite can be susceptible to microbial biofilm formation on its surface, potentially promoting the onset of various infections [1,2].

Another essential material in regenerative medicine is titanium oxide. Titanium oxides are increasingly gaining prominence as coatings for implants due to their unique capacity to foster tissue integration and cell adhesion, which are crucial for successful implantation. These materials are essential in promoting bone healing and facilitating the growth of new bone tissue, thereby significantly ensuring the stability of implants within the patient’s body [3,4]. Rutile coatings specifically improve the surface properties of medical implants, leading to reduced bacterial adhesion and biofilm formation. This effect can potentially lower the risk of infections and alleviate other complications associated with implants [5,6]. Additionally, the surface of titanium oxide can be modified with other elements, such as silver, which are well known for their antibacterial properties. Nanosilver and antibiotics exert comparable effects on microorganisms, yet microorganisms do not typically develop resistance to silver. Silver stands out as one of the most potent elements against a broad spectrum of bacteria and fungi. Its antibacterial action disrupts the cellular structure of bacteria, influences the functions of bacterial enzymes, and damages the cell membrane, culminating in bacterial destruction and inhibiting growth [7]. The careful consideration of silver content is crucial in designing coatings for implants. It is imperative to maintain a relatively low silver content (<1 wt.%) as elevated concentrations have demonstrated increased toxicity, impacting not only bacteria but also human body cells [8,9]. Additionally, silver concentrations below 1 wt.% within Ag-hydroxyapatite coatings on a titanium substrate or in Ag/SiO_2_-β-TCP coatings have been observed to stimulate cell proliferation without inducing cytotoxic effects [10].

Apatites and titanium oxides are predominantly employed as layers for the surface modification of titanium bioalloys. Among these, the NiTi shape memory alloy is extensively utilized in short-term implantology [11]. NiTi alloys with a composition that is near-equiatomic exhibit a distinctive property known as the shape memory effect. However, prolonged implantation of NiTi alloys presents a significant challenge due to the alloy’s high nickel content, posing the risk of releasing toxic ions into the body’s fluids [12]. Addressing this concern involves the surface modification of the alloy by establishing surface layers that act as a mechanical barrier, preventing the release of nickel ions and thereby enabling longer-term implantation. Moreover, these layers can serve multiple additional functions, such as enhancing osseointegration or exhibiting antibacterial properties. For NiTi alloys, the ceramic layers applied to their surface need to be extremely thin to avoid limitation during the induction of the shape memory effect. Thick layers risk being damaged during this process. Therefore, maintaining the minimal thickness of ceramic coatings is essential to preserve the functionality of the shape memory effect in these alloys.

Numerous surface engineering techniques enable the fabrication of thin ceramic layers, particularly those employing nanometric or submicro-metric particles. Within these methods, electrophoretic deposition (EPD) holds significance by offering the capability to produce layers of varying thicknesses and morphologies through the meticulous control of deposition parameters like voltage/current or deposition time [13]. The electrophoresis process shows promise in creating novel materials with intriguing properties, including highly active surfaces that can significantly impact bioactivity and biocompatibility [10].

In this work, the electrophoretic deposition method was employed to develop pioneering multifunctional coatings on a NiTi shape memory alloy. The coatings incorporated a chemically synthesized nanometric molecular system consisting of rutile-silver nanocomposites (Ag-TiO_2_) and hydroxyapatite in submicro- and nanometric grain sizes. The selection of the heat treatment conditions for the deposited layers was based on the outcomes of dilatometry, high-temperature microscopy, and thermogravimetry analyses. The resultant novel coating underwent characterization concerning its structure and morphology, and the mechanism governing layer formation was discussed.

## 2. Materials and Methods

### 2.1. Substrate

The commercially available NiTi alloy, primarily in its β-phase (B2) exhibiting martensitic transformations at temperatures below ambient, served as the substrate material for coating deposition. The samples underwent a polishing process using SiC papers up to a 2000-grit fineness. Preceding the deposition, the sample surfaces were cleansed in acetone within an ultrasonic bath to eliminate residual impurities originating from the preparation process. Subsequently, a passivation process was conducted in an autoclave at 134 °C for a duration of 30 min, with the objective of augmenting corrosion resistance. This passivation method facilitated the formation of a corrosion-resistive, thin, and amorphous TiO_2_ layer, serving as a protective barrier on the surface of the samples [14].

### 2.2. Preparation of Suspension, Electrophoretic Deposition, and Heat Treatment

The hybrid coatings were produced through the electrophoretic deposition (EPD) technique using a colloidal suspension containing a 0.1 wt.% concentration of powders dispersed in 75% ethanol (Avantor Performance Materials, Gliwice, Poland). The Ag-TiO_2_ nanocomposite was prepared according to the procedure described in detail in ref. [15].

Commercially available hydroxyapatite (Sigma-Aldrich, St. Louis, MO, USA), with micro- and nanometric particle sizes, was also used to prepare the colloidal suspension. The powders were mixed at a 1:2 ratio (Ag-TiO_2_: HAp). The suspensions underwent agitation on a magnetic stirrer for 1 h followed by ultrasonic treatment for 2 h.

The suspension’s Zeta potential measured 7.17 ± 0.2 mV, thus enabling cataphoretic deposition to be conducted. The conductivity of the suspension reached 0.223 ± 0.003 mS/cm. The deposition process was executed within a voltage range of 10–30 V and a duration of 1–2 min. Subsequently, the coatings were air-dried at room temperature for 24 h and then subjected to heat treatment at 800 °C under low vacuum conditions for 2 h.

### 2.3. Method of Testing

Using the Malvern Zetasizer Nano ZS particle size analyzer (Malvern Panalytical, Almelo, The Netherlands), the Zeta potential and value and conductivity of the colloidal suspension were measured. The measurement was made three times, and the paper includes the average value along with the standard deviation. The Zeta potential value was determined at room temperature using a U-shaped cuvette (DTS1070 from Malvern).

The scanning electron microscopy (SEM) analysis was conducted using the TESCAN Mira 3 LMU instrument (Brno, Czech Republic), complemented through the use of an energy dispersive spectrometer (EDS) provided by Oxford Instruments–Aztek (Abingdon, UK), to ascertain microstructural properties and conduct chemical analysis. Imaging was achieved by collecting secondary electrons (SEs) and backscattered electrons (BSEs). The specimens under investigation were prepared with a carbon layer using Quorum Q150T ES equipment (Quorum, San Jose, CA, USA). Analysis of the surface chemical composition was performed on five areas of the sample, and then the average was taken.

Simultaneous differential thermal analysis (DTA), thermogravimetric analysis (TG), differential thermogravimetric analysis (DTG), and measurement of gases evolved from the Ag-TiO_2_ nanocomposite (EGA) were performed. The sample was analyzed using a simultaneous STA 409 PC thermal analyzer from NETZSCH and a QMS 403 C Aëolos quadrupole mass spectrometer (NETZSCH, Selb, Germany). A total of 28.1 mg of the sample was placed in an Al_2_O_3_ crucible and heated from 40 °C to 1100 °C at a rate of 20 °C/min with an air flow of 30 mL/min.

The thermal expansion and shrinkage of the powders were assessed using thermo-mechanical analysis (TMA 92 Setaram). TMA experiments were conducted up to 1200 °C with a heating rate of 7 °C/min under an air atmosphere at 1.5 bar. The powder sample was placed in a tiny crucible and loaded using a flat-ended alumina probe with an applied load of 5 g to preserve sample integrity before experimentation. Corrections for blank measurements, specifically the expansion of corundum elements, were applied to the TMA curve.

The investigation of linear changes occurring during the heating process of powders was conducted using a Leitz high-temperature microscope, aiming to simulate the phenomena observed during heat treatment. A manually pressed sample, forming a cube with an approximate side length of 3 mm, was the subject of analysis. Placed upon a corundum pad, the samples underwent heating up to 1200 °C at a rate of 7 °C/min. Utilizing the microscope’s eyepiece, images of the sample were captured at temperature intervals corresponding to changes in its shape. Continuous monitoring and photographic documentation of the sample’s morphological changes and dimensional variations in response to temperature fluctuations facilitated the acquisition of several images that showed the material’s behavior during the heating process. Analysis of these images enabled the determination of the relative change in the sample’s cross-sectional area δ(T) concerning temperature, and the initiation temperature of sintering was determined.

Raman measurements were conducted on NiTi alloy substrates with functionalized surfaces in a mixture of hydroxyapatite particles (HAp) and Ag-TiO_2_ nanocomposites before and after sintering. These measurements were carried out using a WITec confocal alpha 300R Raman microscope manufactured by WITec Wissenschaftliche Instrumente und Technologie GmbH in Ulm, Germany. The microscope was equipped with an air-cooled solid-state laser emitting light at a wavelength of 532 nm and a CCD detector. Laser radiation was directed into the microscope through a polarization-maintaining single-mode optical fiber with a diameter of 50 μm. The scattered radiation was then focused on a multi-mode fiber with a diameter of 50 μm and a 600 line/mm grating monochromator. The spectrometer monochromator was calibrated using the emission lines of a Ne lamp, while the silicon plate’s signal at 520.7 cm^−1^ was used to verify beam alignment.

To ensure optimal parameters between lateral and depth resolution, an Olympus MPLAN 100×/0.9NA objective lens was used (Olympus, Tokyo, Japan) [16]. In this context, lateral resolution (LR) was estimated according to the Rayleigh criterion, which is given through LR = 0.61λ/NA where LR represents the minimum distance between resolvable points in the X- and Y-directions, NA is the numerical aperture, and λ is the wavelength of laser excitation. The result of this calculation was an LR of 0.36 μm. Depth resolution (DR), representing the minimum distance between resolvable points in the Z-direction, was calculated as DR = λ/(NA)², resulting in a DR of 0.65 μm.

A surface Raman imaging map in the X- and Y-directions was collected within a 50 μm × 50 μm area, utilizing 150 × 150 pixels (equivalent to 22,500 spectra). The integration time per spectrum was set to 500 ms, and the sample was moved with a precision of ±0.5 μm during the measurements.

A depth-scan Raman imaging map was created by scanning from +7.0 μm to −7.0 μm in the Z-direction within a 50 μm × 15 μm area. This scan utilized 150 × 45 pixels, resulting in 6750 spectra. The integration time per spectrum remained 500 ms, and the sample was moved with a precision of ±0.5 μm.

All spectra were recorded in the 75–4000 cm^−1^ range, with a laser power of 10 mW on the sample and a spectral resolution of 3 cm^−1^. The acquired data underwent baseline correction using a third-degree auto-polynomial function, and automatic cosmic ray removal was performed.

The basic analysis was carried out using the WITec ProjectFive Plus Software (v5.1.1, WITec Wissenschaftliche Instrumente und Technologie GmbH, Ulm, Germany) to differentiate the chemical and structural characteristics of the coat-forming material. Finally, band fitting analysis was conducted using a Lorentz–Gauss function with the minimum number of components necessary. This analysis was performed on the averaged spectrum obtained from each sample. The entire procedure was repeated five times for a comprehensive NiTi substrate-coating statistical analysis.

## 3. Results and Discussion

### 3.1. Morphological and Structural Characteristics of Deposited Hybrid HAp/Ag-TiO_2_ Coatings

**Figure 1:**
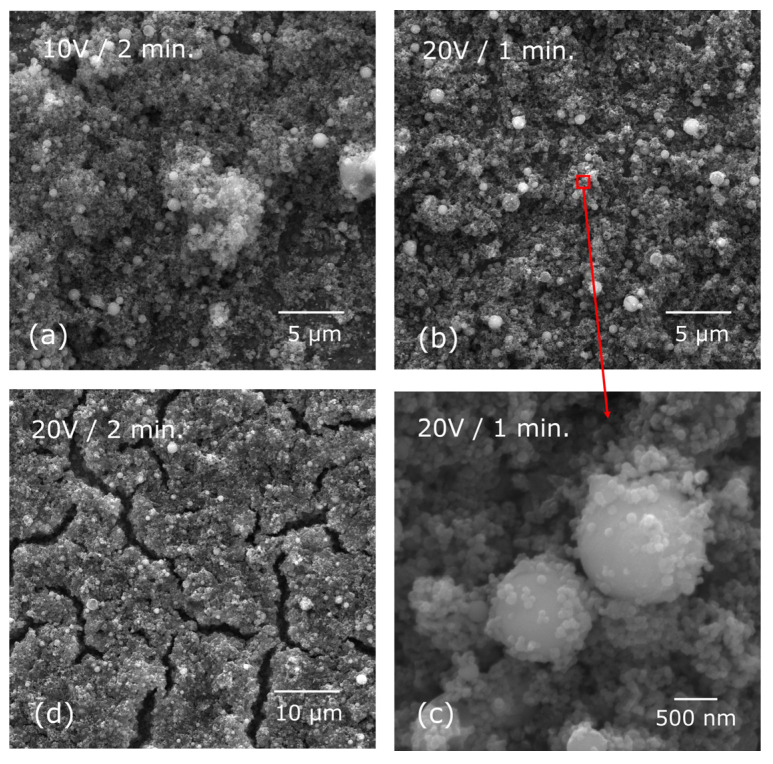
SEM-SE images of the HAp/Ag-TiO_2_ coatings deposited under different conditions: 10 V/2 min. (**a**), 20 V/1 min. (**b**,**c**) and 20 V/2 min (**d**). (**c**) presents a magnified view of the microscopic image marked in (**b**).

Microscopic examinations revealed the significant impact of deposition parameters on the quality of hybrid coatings, which were composed of hydroxyapatite and the Ag-TiO_2_ nanocomposite particles. Layers deposited at a low voltage of 10 V, regardless of the duration (1 or 2 min), exhibited heterogeneity and failed to fully cover the passivated NiTi alloy surface (Figure 1a). Only upon elevating the voltage to 20 V were uniform coatings achieved completely covering the alloy surface (Figure 1b). Microscopic observations showed the mixing of various coating components resulting in a composite structure. Figure 1 illustrates that larger spherical hydroxyapatite particles are embedded in a finer fraction, encompassing both hydroxyapatite (spherical particles) and Ag-TiO_2_ nanocomposite (irregularly shaped particles). Additionally, Figure 1c displays the deposition of a finer composition onto the surface of larger particles. It was found that extending the deposition time resulted in overly thick coatings prone to cracking during ambient drying (Figure 1d). A similar outcome was observed from an increased voltage (30 V) for both 1 min and 2 min deposition times. The presence of imperfections, particularly cracks following the deposition process, renders the layers unsuitable for medical applications. Therefore, only the layers obtained under the condition of 20 V/1 min were considered for subsequent research.

**Figure 2:**
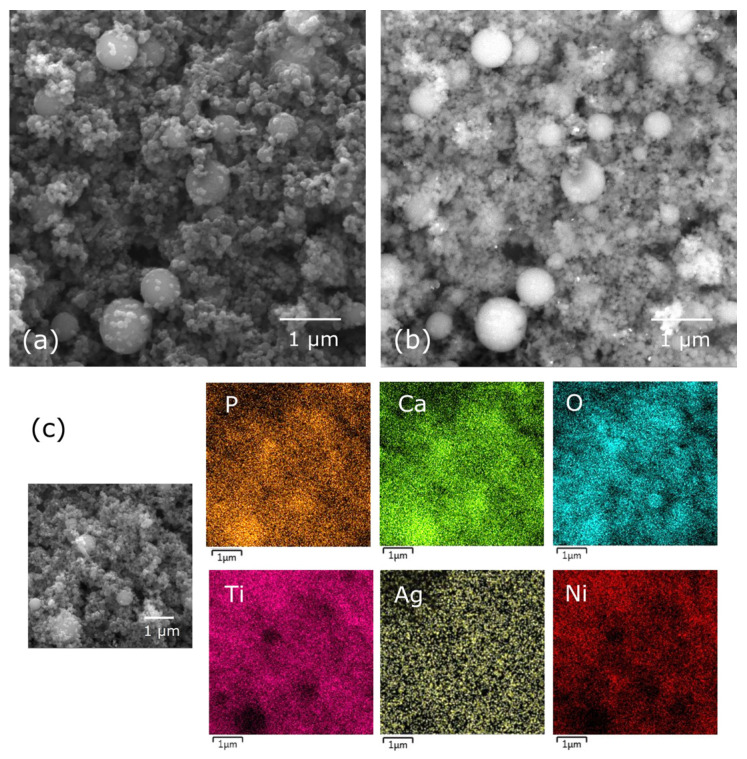
SEM-SE image (**a**), SEM-BSE image (**b**), and element distribution map (**c**) of the coating HAp/Ag-TiO_2_ (20 V/1 min).

Analysis of the SEM-BSE images and elemental distribution maps (Figure 2) yielded crucial insights into the dispersion of silver particles within the coating. The distribution and silver content hold significance concerning the material’s antibacterial properties. Analysis indicated a dispersed presence of silver throughout the coating, yet tendencies toward agglomeration or the presence of larger particles (identified as the brighter dots in Figure 2b) were observed. Chemical composition analysis unveiled a silver content of 1.1 ± 0.2 wt.% within the layer. Literature references emphasize that in coatings designed for medical applications, maintaining a relatively low silver content (not surpassing 1 wt.%) is imperative to prevent potential cytotoxic effects on human body cells [9,10]. Furthermore, chemical composition analysis highlighted the presence of elements constituting hydroxyapatite (calcium, phosphorus, oxygen) and titanium—a constituent of both the Ag-TiO_2_ nanocomposite and the metallic substrate. The nickel signal indicated that a relatively thin layer was formed. Element distribution maps confirm that the individual components of the coating are mixed and form a composite layer.

**Figure 3:**
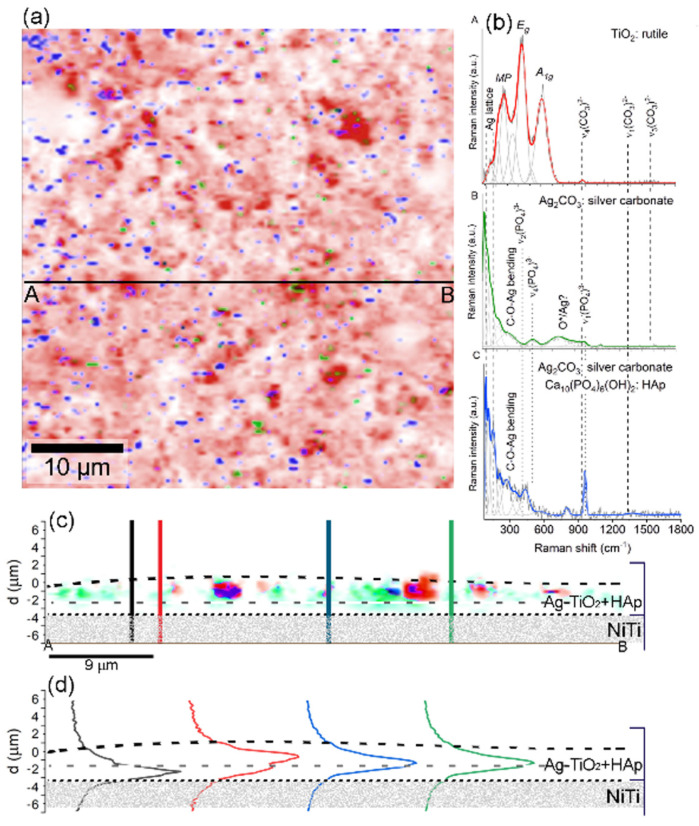
After deposition, a chemical and structural differentiation image of the HAp/Ag-TiO_2_/NiTi surface is depicted in the X- and Y-directions (**a**) and the X- and Z-directions (**c**) along the line A-B. The individual color-highlighted phases on the Raman maps were summarized as the averaged Raman spectra (**b**). At the same time, depth-scan profiles for four exemplary locations along the X- and Y-cross lines were presented in panel (**d**). Dashed lines on the depth profiles delineate the boundary of the HAp/Ag-TiO_2_ layer.

Raman spectroscopy played a crucial role in verifying the modification of the NiTi surface, including an extensive overview of the chemical and structural phase diversity throughout the entire HAp/Ag-TiO_2_ coating. Table S1 presents the assignment of all Raman bands based on the collected spectra. An analysis of the X–Y Raman maps has revealed a complex three-phase system with dominant coat-forming titanium dioxide along with irregularly dispersed tiny particles forming low-dimensional clusters consisting of a mixture of silver carbonate and hydroxyapatite. Rutile uniformly covers the NiTi surface with variable aggregation densities, which is evident when looking at the color intensity across the coating (brighter and darker red areas in Figure 3a).

A closer examination (Figure 3b) shows that titanium dioxide is characterized by a rutile-related band arrangement with maxima located at 268 cm^−1^ (indicative of multiple-phonon scattering processes), 431 cm^−1^ (E_g_ symmetry), and 606 cm^−1^ (A_1g_ symmetry), with the E_g_ mode significantly downshifted compared with typical literature-derived band positions [17,18]. According to the literature, the downshift of the E_g_ mode, which is attributed to in-plane vibrations of O-O bonds, results from a lower O/Ti ratio [19,20], thereby leading to an increase in oxygen vacancies in TiO_2_ and structural distortions of titanium octahedra. The observable distortion supports modifying the rutile structure during the synthesis of the Ag-TiO_2_ system. The distortion of titanium octahedra, on the one hand, enforces the formation of Ag-O-Ti interconnections [21]. On the other hand, the presence of a highly reactive chemical environment and the incorporation of atmospheric carbon dioxide into the solution lead to the formation of Ti-O-C bonds. This, coupled with the high accessibility of silver ions in the solution and their affinity for carbon dioxide, favors the formation of aragonite structure silver carbonates. These are evidenced by bands centered around 90, 120, 150, 200–300 cm^−1^ (indicating silver lattice vibrational modes), and 1380–1560 cm^−1^ (ν_1,3_(CO_3_)^2−^) [15,22]. Furthermore, molecularly chemisorbed oxygen species found around some silver carbonates are evidenced by the band at 760 cm^−1^ [23,24,25,26].

A low Raman signal indicates the presence of tiny hydroxyapatite particles. However, characteristic hydroxyapatite-related bands around 961 and 958 cm^−1^, stemming from symmetric stretching vibrations within ν_1_(PO_4_)^3−^, are still discernible [27,28,29].

According to the literature, the slight shift towards lower frequencies results from structural modifications due to the interaction with the mobile ionic form of Ag suspended in the colloidal suspension, which is comparable to the available literature data. A similar effect was observed in the case of Ag-SiO_2_ + HAp/TiO_2_/NiTi [30] or Ag-SiO_2_ + β-TCP/TiO_2_/NiTi composite systems [10]. Other bands generally exhibit low intensity and overlap with the more intense silver carbonate bands (Figure 3b).

The fundamental analysis of the Raman cross-sections in the X–Z direction revealed rutile and silver carbonate distribution throughout the coating (Figure 3b,c). Rutile particles formed smaller, irregular clusters, while silver carbonate particles were more uniformly distributed closer to the NiTi substrate. Hydroxyapatite particles tended to accumulate heterogeneously, which often occurred around the rutile agglomerates (Figure 1c). This analysis indicated a two-stage coating formation process. Initially, a thinner layer composed of Ag_2_CO_3_ and Ag-TiO_2_ nanocomposites, which were approximately 1.5 μm thick, was formed near the NiTi surface during the electrophoretic deposition (Figure 3d). Subsequently, with the continued deposition process, tiny rutile and hydroxyapatite particles aggregated, thereby giving the coating a more textured appearance and a thickness ranging from about 1.8 μm to 2.2 μm (Figure 3d).

### 3.2. Determination of the Heat-Treatment Temperature of HAp/Ag-TiO_2_ Coatings

The heat treatment of electrophoretically deposited ceramic layers is critical in establishing a chemical bond between the coating and the metallic substrate [31]. This stage also induces the sintering of ceramic particles. Additionally, the temperature factor significantly influences the thermal expansion of the ceramic layers, contributing to their contraction and the consequent generation of internal stresses within the material. These stresses can lead to the formation of cracks and the delamination of coatings [30]. Thus, it becomes crucial to ascertain characteristic temperatures and understand the behavior of ceramics intended for use as coating materials. Dilatometric and high-temperature microscope tests were conducted on coat-forming powders to fulfill this objective.

**Figure 4:**
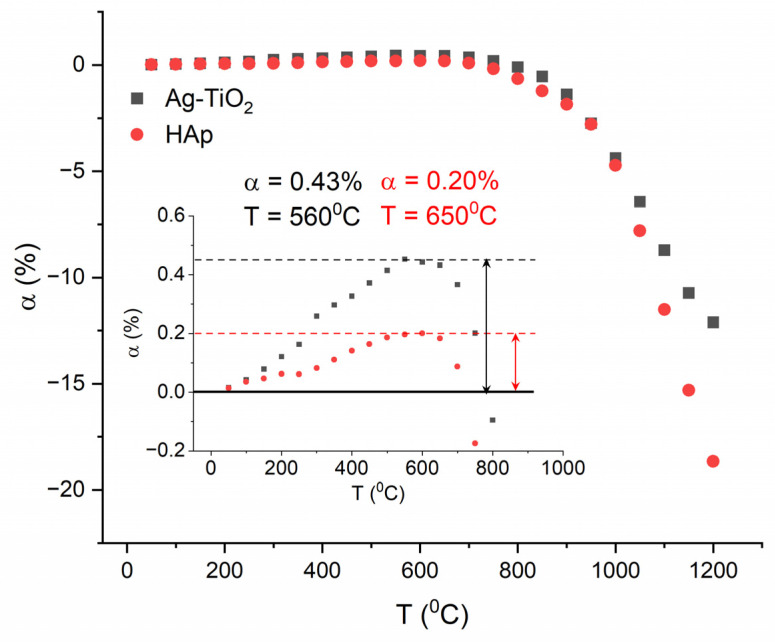
Change in the linear dimensions of the Ag-TiO_2_ nanocomposite and hydroxyapatite powders at different temperatures.

When analyzing the linear changes exhibited by the coat-forming nanopowders at different temperatures, a notable similarity in the course of the curve was observed (Figure 4). Both the hydroxyapatite and the Ag-TiO_2_ nanocomposite showed low thermal expansion. Hydroxyapatite exhibits this feature up to a temperature of approximately 650 °C (α = 0.20%), while the nanocomposite tends to expand up to a temperature of approximately 560 °C (α = 0.43%). The shrinkage behavior in both materials, indicative of the onset of sintering, initiates at around 800 °C and increases significantly from approximately 1000 °C.

**Figure 5:**
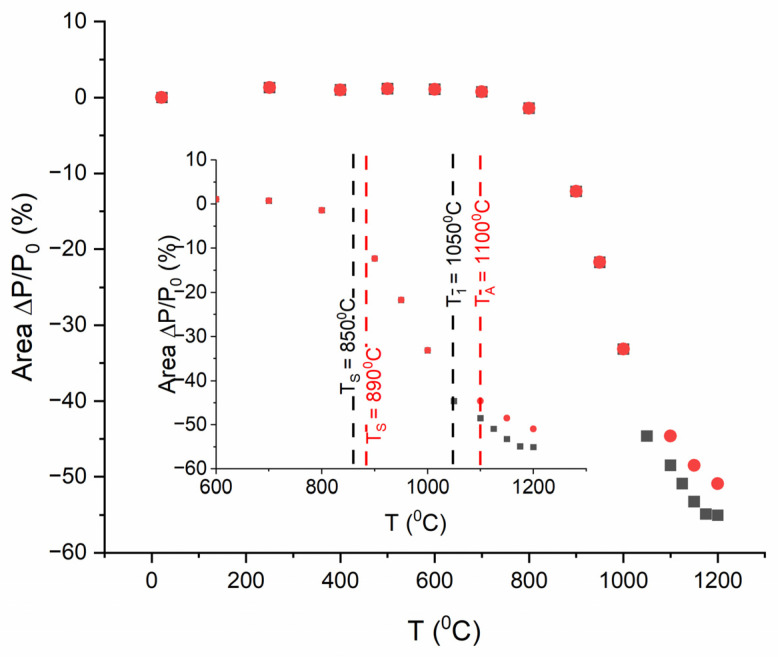
Dimensional changes observed for the Ag-TiO_2_ nanocomposite (black marks) and hydroxyapatite powders (red dots), where T_s_ is the sintering start temperature and T_1_ is the end of sintering temperature.

Studies using a high-temperature microscope (Figure 5) revealed that the change in the cross-sectional area of both the HAp and Ag-TiO_2_ samples commence above 800 °C, with the onset of sintering identified at 890 °C and 850 °C for hydroxyapatite and the nanocomposite, respectively. Within the temperature range of 890–1100 °C for HAp and 850–1050 °C for the Ag-TiO_2_ nanocomposite, a linear shift in the sample’s cross-sectional area was observed. At a temperature of 1100 °C for hydroxyapatite and 1050 °C for the nanocomposite, a softening point occurred, which was associated with the appearance of a small amount of liquid phase at the grain boundary. The maximal shrinkage from sintering initiation to the softening temperature was measured at 9% for HAp and 7% for Ag-TiO_2_. The most substantial alteration in the cross-sectional area of the HAp sample reached 18% at 1200 °C, while it was notably higher in the case of the composite at 55%.

**Figure 6:**
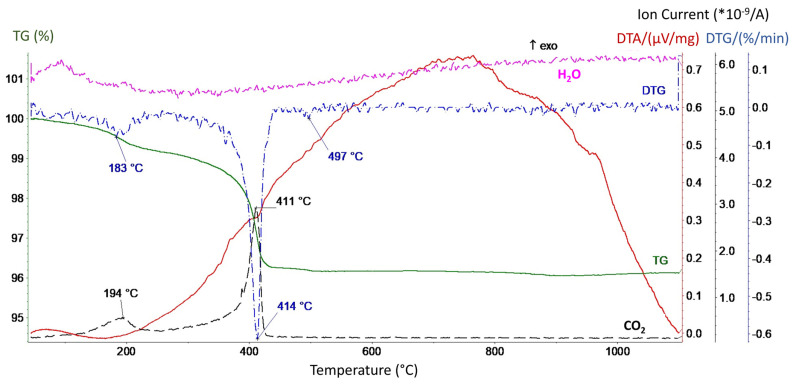
DTA/TGA/EGA curves for the Ag-TiO_2_ nanopowder.

Understanding the thermal stability of materials holds significant importance when determining the appropriate heat treatment temperature. Thus, the thermogravimetric method determined the thermal stability of the Ag-TiO_2_ nanopowder concerning changeable temperature conditions (Figure 6). Until approximately 525 °C, the sample underwent a 3.85% reduction in its initial mass occurring in two distinct stages as follows: an initial loss of 0.90% up to 285 °C, followed by a subsequent loss of 2.95% up to 525 °C. This phenomenon primarily arises from the release of small amounts of physically bound water, which reaches its maximum release at approximately 94 °C, and the emission of carbon dioxide from the decomposition of silver carbonates. Notably, the highest emission of CO_2_ is observed at temperatures of 194 °C and 411 °C.

The thermal stability of the second coating component, hydroxyapatite, has been extensively described in the literature [10]. Previous research findings indicate that with rising temperatures, there is a gradual release of water molecules from hydroxyapatite. This phenomenon correlates with absorbed water at temperatures of up to approximately 350 °C. The porous nature of HAp leads to a stronger binding of sorbed water within its structure, thereby elongating the process of its release and necessitating higher temperatures. Experimental evidence has demonstrated that around 800 °C, the gradual dehydroxylation of HAp initiates due to the loss of constitutional water. This process notably intensifies at 1171 °C.

Considerations regarding the metallic substrate and its response to high temperatures are crucial when determining the parameters for heat treatment. In the context of NiTi shape memory alloys, subjecting them to high temperatures can trigger the decomposition of the alloy into equilibrium and no-equilibrium phases. This in turn reduces transformation enthalpy and detrimentally impacts the shape memory effect. Consequently, heat treatments for layers on such alloys are limited to a maximum temperature of 900 °C. In the case of NiTi alloys, the utilization of a protective atmosphere or vacuum conditions are pivotal. These conditions are essential to safeguard the alloy against unwanted oxidation or other detrimental reactions during heat treatment processes [32].

Considering the behavior of coat-forming materials at elevated temperatures, their dimensional changes, thermal stability, and the known heat treatment temperatures for NiTi alloys from the literature, it is advisable to limit the annealing temperature of the deposited layers to 800 °C. Consequently, the HAp/Ag-TiO_2_ layers, deposited electrophoretically at 20 V for 1 min, underwent heat treatment at 800 °C for 2 h under low vacuum conditions.

### 3.3. Morphological and Structural Characteristics of Heat-Treated HAp/Ag-TiO_2_ Coatings

**Figure 7:**
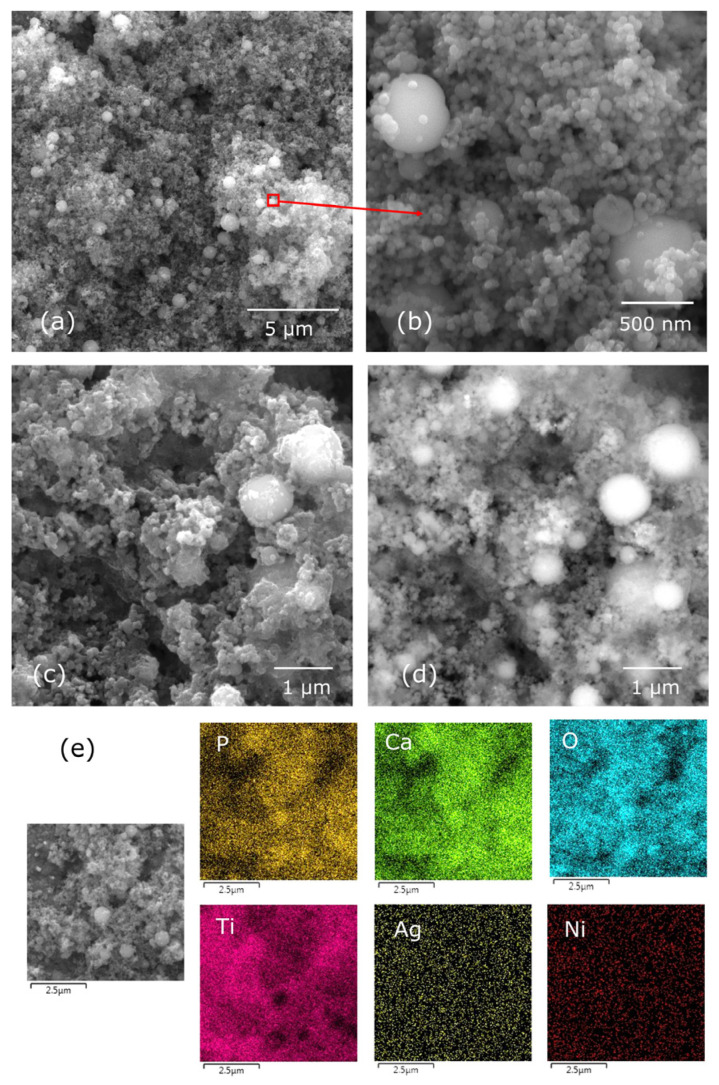
SEM-SE image (**a**–**c**), SEM-BSE image (**d**), and element distribution map (**e**) of the coating HAp/Ag-TiO_2_ after heat treatment. (**b**) presents a magnified view of the microscopic image marked in (**a**).

The applied heat treatment parameters, as observed through microscopic examinations, did not induce significant changes in the morphology of the deposited coatings (Figure 7). No evidence of cracks or layer delamination was noted. Under higher magnification, it became apparent that the layer had attained a more compact structure compared with its state immediately after electrophoretic deposition (Figure 7b,c), which was potentially due to the localized melting of the ultrafine fraction. Analysis of BSE-SEM images (Figure 7d) revealed that heat treatment led to the decomposition of larger silver particles or agglomerates, which are visible in Figure 2b as bright spots. Nonetheless, fine silver particles remained identifiable and uniformly dispersed throughout the coating (Figure 7e). The chemical composition analysis indicated a small decrease in the silver content to approximately 0.8 ± 0.2 wt.%. Raman studies (Figure 3) displayed that the silver in the deposited coating was partly bound to the carbonates, which gradually decomposed (Figure 6). This decomposition led to the partial evaporation of the smallest silver fraction along with the disintegration of agglomerates, thus contributing to the reduced silver content. The chemical composition analysis additionally revealed the presence and content of several elements within the coating as follows: phosphorus (P), oxygen (O), and calcium (Ca) from the hydroxyapatite particles; titanium (Ti) from the nanocomposite and the substrate; and nickel (Ni) originating from the substrate.

The heat treatment applied led to structural modifications within the layer. Raman imaging maps, which were acquired from diverse areas spanning the entire coating, were meticulously analyzed in both the X- and Y-directions to discern spatial variations in the phase compositions of the materials. Concurrently, an X- and Z-direction examination was conducted to elucidate the mechanisms governing coating formation after heat treatment. Table S1 presents the assignment of Raman bands based on the collected spectra.

**Figure 8:**
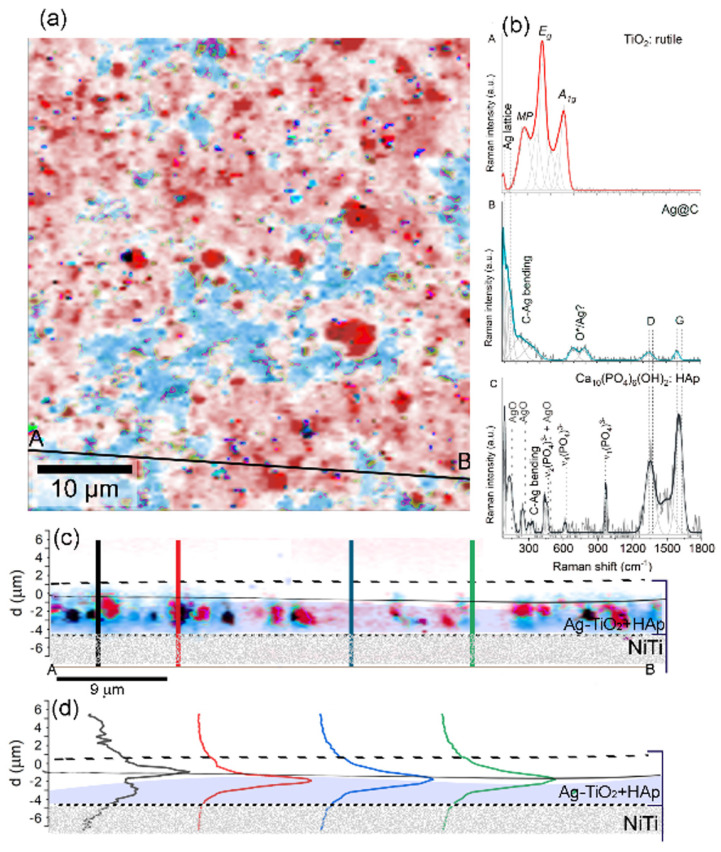
After the sintering, a chemical and structural differentiation image of the functionalized HAp/Ag-TiO_2_/NiTi surface was depicted in the X- and Y-directions (**a**) and the X- and Z-directions (**c**) along the line A-B. The individual color-highlighted phases on the Raman maps were summarized in the averaged Raman spectra in (**b**). At the same time, depth-scan profiles for four exemplary locations along the X- and Y-cross lines were presented in panel (**d**). Dashed lines on the depth profiles delineate the boundary of the HAp/Ag-TiO_2_ layer.

After heat treatment, rutile remained stable, exhibiting only insignificant band shifts towards 268 cm^−1^ (multiple-phonon scattering processes), 428 cm^−1^ (E_g_ symmetry), and 612 cm^−1^ (A_1g_ symmetry) (Figure 8b) [17,18]. These shifts indicated an increase in oxygen vacancies and structural distortions of titanium octahedra probably as a result of the temperature-induced decomposition of Ag-O-Ti interconnections that led to the release of silver oxides [20]. A similar transformation was expected for the Ti-O-C interconnections, which gradually degraded as the temperature increased. According to the literature, this degradation led to the conversion of the initial carbonates into low-temperature phases, with a positive expansion of the unit cell in the a- and c-axes [33], silver oxide (ca. 230 °C), and finally metallic silver (ca. 380 °C) with the diffusional removal of CO_2_ through the surface product layer [34]. Based on the results of thermogravimetric tests, the temperatures of these transformations in the Ag-TiO_2_ nanocomposite were altered. The initial reaction occurred at approximately 194 °C, while the subsequent one took place at around 411 °C. It seemed that remnants after the decomposition of carbon dioxide in such a context were entrapped within the silver nanoparticle cores, forming a layered carbon system with unsaturated bonds (Figure 8b). This led to the activation of bands between 200 and 400 cm^−1^ (C-Ag bending) and around 1340 and 1580 cm^−1^ (D- and G-planes) [35,36]. Raman chemical maps after sintering unveiled the coexistence of TiO_2_ with Ag@C (Figure 8b). Confirmation of the metallic silver particle formation led to the appearance of bands around 610, 704, and 808 cm^−1^, which indicated that molecularly chemisorbed oxygen species (Oβ, OH) had formed around the silver [10,11,12,13]. Raman spectroscopy confirmed the presence of highly stable hydroxyapatite, as evidenced by characteristic bands found in the range between 969 and 963 cm^−1^ (ν_1_(PO_4_)^3−^), 596 cm^−1^ (ν_4_(PO_4_)^3−^), 446 cm^−1^ (ν_2_(PO_4_)^3−^), and in the range between 350 and 150 cm^−1^ (lattice modes of Ca(PO_4_)) [14,15,16]. Hydroxyapatite bands coexisted with silver oxide bands located at 133, 243, and 440 cm^−1^ (Figure 8b) [23] and probably shield silver oxide from complete decomposition at the proposed sintering temperature. The decomposition of silver carbonates also provided extra dynamically moving and reactive carbon dioxide molecules that tend to be incorporated into the hydroxyapatite structure, thereby forming a carbonate apatite (CHAp). Such structural modification correlates with highly intense and broad Raman bands appearing in the 1550–1280 cm^−1^ region, often called G- and D-bands. A similar effect has been observed for hydroxyapatite coatings sintered under similar thermodynamic conditions [14].

Consistent with the as-prepared composites, post-sintering Raman maps, based on basis analyses in the X–Y directions and cross-sections in the X–Z direction, revealed a complex three-phase system that uniformly coats the NiTi substrate but displays a strong tendency to segregate. As a result, the coat-forming material is composed of a mixture of titanium dioxide and carbonaceous phases uniformly covered by the NiTi substrate with irregularly dispersed hydroxyapatite particles. Thermal-induced reorganization of titanium dioxide particles results in heterogeneous distribution, aggregation, and consolidation into irregular structures (Figure 8a,b). In turn, silver-carbonaceous structures are free-volume coat-forming fillers that are unevenly distributed and display irregularly shaped structures (Figure 8b,c). Finally, the thickness, estimated by calculating the full width at the half maximum value of the Raman signal, indicates two-layered systems, each with a thickness of approximately 2 μm (Figure 8d).

## 4. Conclusions

The primary aim of this study was the functionalization of the NiTi shape memory alloy surface, thereby enabling its prolonged use in implant medicine. To achieve this goal, innovative multifunctional hybrid layers composed of a nanometric molecular system combining silver-rutile (Ag-TiO_2_), which is renowned for its antibacterial properties, with bioactive submicro- and nanosized hydroxyapatite (HAp) were produced. The multifunctional coatings were deposited using the electrophoretic deposition method (EPD) with varying voltages (ranging from 10 to 30 V) and deposition times (from 1 to 2 min), achieving a continuous, crack-free coating at 20 V/1 min. The silver present within the layer existed in the form of nanometric silver carbonates (Ag_2_CO_3_) and metallic nanosilver. The components of the coating with rutile and hydroxyapatite formed a composite layer. Subsequently, based on analyses from DTA/TG, dilatometric tests, and high-temperature microscopy, the heat treatment temperature for the deposited layers was set at 800 °C for 2 h. These procedures generated a novel material exhibiting a distinct structure compared with the initial nanopowders. The resultant composite layer contained hydroxyapatite (HAp), apatite carbonate (CHAp), metallic silver, silver oxides, Ag@C, and rutile with a defective structure, thereby significantly enhancing its activity and influencing both its bioactivity and biocompatibility properties. Notably, the produced layers, measuring 2 μm in thickness, exhibited a crack-free nature without any signs of delamination.

In summary, the applied processes yielded an exceptionally reactive layer on the NiTi alloy, holding significant promise in implantation medicine. However, further extensive research is essential to assess the efficacy, longevity, and safety of these coatings across diverse medical applications. The coatings produced have undergone a comprehensive characterization process, encompassing assessments of corrosion mechanisms, bioactivity, wettability, biocompatibility, cytotoxicity, and microbiological aspects. The findings from these tests will be included in the forthcoming publication, providing detailed insight into the performance and potential applications of these coatings.

## Data Availability

The data presented in this study are openly available in RepOD https://repod.icm.edu.pl/dataset.xhtml?persistentId=doi:10.18150/64KWUO (accessed on 17 December 2023).

## Supplementary Materials

The following supporting information can be downloaded at: https://www.mdpi.com/article/10.3390/ma17030604/s1, Table S1: Raman band assignment based on the spectra gathered before and after the sintering procedure.

## References

[1] Chen X., Li H., Ma Y., Jiang Y. (2023). Calcium Phosphate-Based Nanomaterials: Preparation, Multifunction, and Application for Bone Tissue Engineering. Molecules.

[2] Dorozhkin S.V. (2015). Calcium orthophosphate deposits: Preparation, properties and biomedical applications. Mater. Sci. Eng. C Mater. Biol. Appl..

[3] Santiago-Medina P., Sundaram P.A., Diffoot-Carlo N. (2015). Titanium oxide: A bioactive factor in osteoblast differentiation. Int. J. Dent..

[4] Chouirfa H., Bouloussa H., Migonney V., Falentin-Daudré C. (2019). Review of titanium surface modification techniques and coatings for antibacterial applications. Acta Biomater..

[5] Vieira A., Rodríguez-Lorenzo L., Leonor I.B., Reis R.L., Espiña B., Barreiros dos Santos M. (2023). Innovative Antibacterial, Photocatalytic, Titanium Dioxide Microstructured Surfaces Based on Bacterial Adhesion Enhancement. ACS Appl. Bio Mater..

[6] Jia L., Qiu J., Du L., Li Z., Liu H., Ge S. (2017). TiO_2_ Nanorod Arrays as a Photocatalytic Coating Enhanced Antifungal and Antibacterial Efficiency of Ti Substrates. Nanomedicine.

[7] Ge L., Li Q., Wang M., Ouyang J., Li X., Xing M.M. (2014). Nanosilver particles in medical applications: Synthesis, performance, and toxicity. Int. J. Nanomed..

[8] Morozova V.O., Klinov V.D. (2021). Nanosilver in Biomedicine: Advantages and Restrictions. Silver Micro-Nanoparticles—Properties, Synthesis, Characterization, and Applications.

[9] Djokić S. (2016). Biomedical and Pharmaceutical Applications of Electrochemistry.

[10] Dulski M., Dudek K., Chalon D., Kubacki J., Sulowicz S., Piotrowska-Seget Z., Mrozek-Wilczkiewicz A., Gawecki R., Nowak A. (2019). Toward the development of an innovative implant: NiTi alloy functionalized by multifunctional β-TCP+Ag/SiO_2_ coatings. ACS Appl. Bio Mater.

[11] Yoneyama T., Miyazaki S. (2008). Shape Memory Alloys for Biomedical Applications.

[12] Shabalovskaya S., Anderegg J., Van Humbeeckm J. (2008). Critical overview of Nitinol surfaces and their modifications for medical applications. Acta Biomater..

[13] Corni I., Ryan M.P., Boccaccini A.R. (2008). Electrophoretic deposition: From traditional ceramics to nanotechnology. J. Eur. Ceram. Soc..

[14] Łosiewicz B., Popczyk M., Goryczka T., Lelątko J., Smołka A., Kowalski P. (2013). Structure and Resistance to Electrochemical Corrosion of NiTi Alloy. Solid State Phenom..

[15] Dudek K., Dulski M., Podwórny J., Kujawa M., Rawicka P. (2023). Optimi-zation of the Electrophoretic Deposition Parameters and Mechanism of Formation of Ag-TiO_2_ Nano-coating on a NiTi Shape Memory Alloy: Part I. Coatings.

[16] Hollricher O., Ibach W. (2010). High-Resolution Optical and Confocal Microscopy. Confocal Raman Microscopy.

[17] Mazza T., Barborini E., Piseri P., Milani P. (2007). Raman spectroscopy characterization of TiO_2_ rutile nanocrystals. Phys. Rev. B Condens. Matter Mater. Phys..

[18] Porto S.P., Fleury P.A., Damen T.C. (1967). Raman Spectra of TiO_2_, MgF_2_, ZnF_2_, FeF_2_, and MnF_2_. Phys. Rev. B.

[19] Kernazhitsky L., Shymanovska V., Gavrilko T., Naumov V., Fedorenko L., Kshnyakin V., Baran J. (2018). Laser-Excited Excitonic Luminescence of Nanocrystalline TiO_2_ Powder. Ukr. J. Phys..

[20] Parker J.C., Siegel R.W. (1990). Calibration of the Raman spectrum to the oxygen stoichiometry of nanophase TiO_2_. Appl. Phys. Lett..

[21] Zahornyi M.M., Tyschenko N.I., Lobunet T.F., Kolomys O.F., Strelchuk V.V., Naumenko K.S., Biliavska L.O., Zahorodnia S.D., Lavrynenko O.M., Ievtushenko A.I. (2021). The Effect of Ag Content on the Structural, Optical, and Cytotoxicity Properties of TiO_2_ Nanopowders Grown from TiO(OH)_2_ Precursor by the Chemical Deposition Method. J. Nano-Electron. Phys..

[22] Martína I., Wiesinger R., Schreiner M. (2012). Micro-Raman Characterisation of Silver Corrosion Products: Instrumental Set Up and Reference Database. E-Preserv. Sci..

[23] Bao X., Muhler M., Pettinger B., Schlögl R. (1993). On the nature of the active state of silver during catalytic oxidation of methanol. Catal. Lett..

[24] Millar G.J., Metson J.B., Bowmaker G.A., Cooney R.P. (1995). In situ Raman studies of the selective oxidation of methanol to formaldehyde and ethene to ethylene oxide on a polycrystalline silver catalyst. J. Chem. Soc..

[25] Pettinger B., Bao X., Wilcock I.C., Muhler M., Ertl G. (1994). Surface-enhanced Raman scattering from surface and subsurface oxygen species at microscopically well-defined Ag surfaces. Phys. Rev. Lett..

[26] Bao X., Muhler M., Pettinger B., Uchida Y., Lehmpful G., Schlögl R., Ertl G. (1995). The effect of water on the formation of strongly bound oxygen on silver surfaces. Catal. Lett..

[27] De Aza P.N., Guitian F., Santos C., de Aza S., Cusco R., Artus L. (1997). Vibrational Properties of Calcium Phosphate Compounds. 2. Comparison between Hydroxyapatite and β-Tricalcium Phosphate. Chem. Mater..

[28] Cusco R., Guitian F., de Aza S., Artus L. (1998). Differentiation between Hydroxyapatite and β-Tricalcium Phosphate by Means of μ-Raman Spectroscopy. J. Eur. Ceram. Soc..

[29] Dudek K., Dulski M., Goryczka T., Gerle A. (2018). Structural changes of hydroxyapatite coating electrophoretically deposited on NiTi shape memory alloy. Ceram. Int..

[30] Dudek K., Dulski M., Łosiewicz B. (2020). Functionalization of the NiTi Shape Memory Alloy Surface by HAp/SiO_2_/Ag Hybrid Coatings Formed on SiO_2_-TiO_2_ Glass Interlayer. Materials.

[31] Zhitomirsky I. (2002). Cathodic electrodeposition of ceramic and organoceramic materials. Fundamental aspects. Adv. Colloid Interface.

[32] Goryczka T., Dudek K., Lelątko J., Wierzchoń T. (2015). Martensitic transformation in NiTi alloy covered by protective multi-layers. Mater. Today Proc..

[33] Norby P., Dinnebier R., Fitch A.N. (2002). Decomposition of Silver Carbonate—The Crystal Structure of Two High-Temperature Modifications of Ag_2_CO_3_. Inorg. Chem..

[34] Waterhouse G.I.N., Bowmaker G.A., Metson J.B. (2001). The thermal decomposition of silver (I, III) oxide: A combined XRD, FT-IR and Raman spectroscopic study. Phys. Chem. Chem. Phys..

[35] Zhang D., Zhang C., Liu J., Chen Q., Zhu X., Liang C. (2019). Carbon-Encapsulated Metal/Metal Carbide/Metal Oxide Core–Shell Nanostructures Generated by Laser Ablation of Metals in Organic Solvents. ACS Appl. Nano Mater..

[36] Li J.G., Tsai C.Y., Kuo S.W. (2014). Fabrication and Characterization of Inorganic Silver and Palladium Nanostructures within Hexagonal Cylindrical Channels of Mesoporous Carbon. Polymers.

