# Supplementary material for: Functionalization of the NiTi Shape Memory Alloy Surface through Innovative Hydroxyapatite/Ag-TiO2 Hybrid Coatings
Source: Materials (Basel). 2024 Jan 26;17(3):604. doi: 10.3390/ma17030604 (PMC10856126; doi:10.3390/ma17030604)
Supplement: Supplementary file 1 [file materials-17-00604-s001.zip › materials-2805775-supplementary.pdf]

materials

MDPI

Supplementary Materials

Functionalization of the NiTi Shape Memory Alloy Surface by Innovative Hydroxyapatite/Ag-TiO_2 Hybrid Coatings

Karolina Dudek [1,*], Mateusz Dulski [2,*], Jacek Podwórny [1], Magdalena Kujawa [1], Anna Gerle [1] and Patrycja Rawicka [3]

1 Łukasiewicz Research Network–Institute of Ceramics and Building Materials, Cementowa 8, 31-983 Kraków, Poland; jacek.poworny@icimb.lukasiewicz.gov.pl (J.P.); magdalena.kujawa@icimb.lukasiewicz.gov.pl (M.K.); anna.gerle@icimb.lukasiewicz.gov.pl (A.G.)
2 Institute of Materials Engineering, University of Silesia, 75 Pułku Piechoty 1A, 41-500 Chorzów, Poland
3 Institute of Physics, Faculty of Science and Technology, University of Silesia in Katowice, 75 Pułku Piechoty 1a, 41-500 Chorzów, Poland; patrycja.rawicka@us.edu.pl
* Correspondence: karolina.dudek@icimb.lukasiewicz.gov.pl (K.D.), mateusz.dulski@us.edu.pl (M.D.)

Table S1. Raman band assignment based on the spectra gathered before and after the sintering procedure.

Initial				After sintering			
ν (cm^{-1})	ν (cm^{-1})	ν (cm^{-1})	Assignement	ν (cm^{-1})	ν (cm^{-1})	ν (cm^{-1})	Assignement
90	110	150	Ag lattice		122	133	Ag lattice/AgO/Ca(PO_4)
120	146	208			154	243	AgO/Ca(PO_4)
150	210	269			222		Ag–O–Ti/Ca(PO_4)
212			Ag–O–Ti				
270	289		multiple-phonon scattering processes	268			defects
344		337	defects	371	322	323	C-Ag
431		430	E_g	428		440	
						446	$\nu_2(PO_4)^{3-}$
513	520		defects	507			
			defects	560			
606			A_{1g}/ $\nu_4(PO_4)^{3-}$	612		596	$\nu_4(PO_4)^{3-}$
					697		chemisorbed oxygen species
	760	798	chemisorbed oxygen species		792		
	895						
971	963	969	$\nu_4(CO_3)^{2-}$ / $\nu_1(PO_4)^{3-}$*			958	$\nu_1(PO_4)^{3-}$
1398		1378	$\nu_1(CO_3)^{2-}$		1340	1351	D
						1483	
1577			$\nu_3(CO_3)^{2-}$		1580	1604	G
